# Supplementary material for: Transposable Element Landscape in the Monotypic Species Barthea barthei (Hance) Krass (Melastomataceae) and Its Role in Ecological Adaptation
Source: Biomolecules. 2025 Feb 27;15(3):346. doi: 10.3390/biom15030346 (PMC11939994; doi:10.3390/biom15030346)
Supplement: Supplementary file 1 [file biomolecules-15-00346-s001.zip › Supplementary_Figures_Barthea_TE_revised1.pdf]

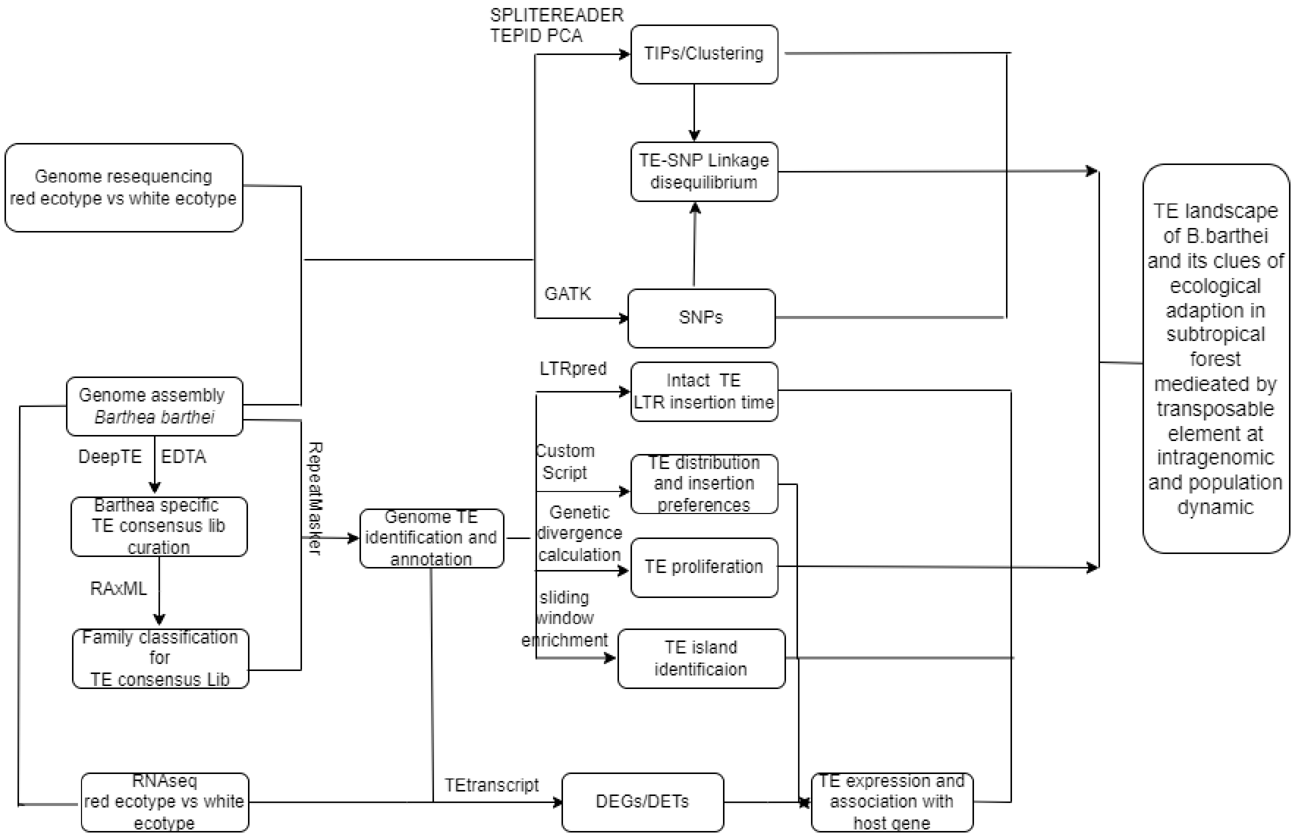

Figure S1. Analytical pipeline for transposable element dynamics and ecological adaptation in *Barthea barthe*i.

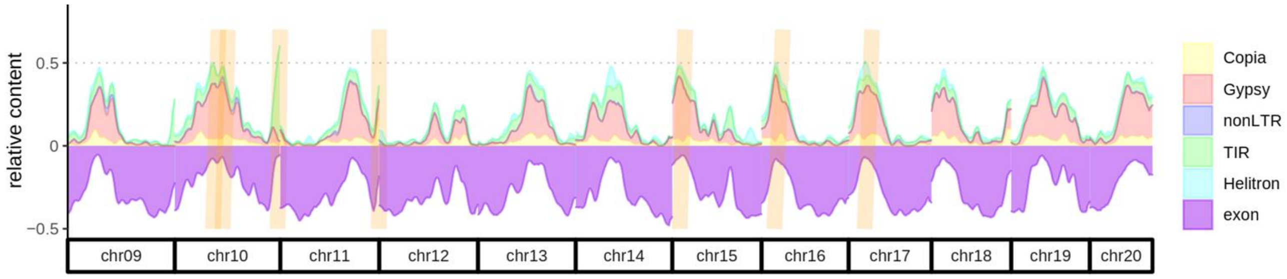

Figure S2. The distribution of transposable element landscapes shown for the genome assembly of *Bathea barthei* across the chromosomes between chr09 and chr20 (continued from Figure 1F). the relative content of different orders or superfamilies of transposable elements or host exons represented by corresponding color, and transposable element islands highlighted with orange bar, the superfamilies/genes including Copia (yellow), Gypsy (red), non-LTR (purple), TIR (green), Helitron (cyan), exon (magenta).

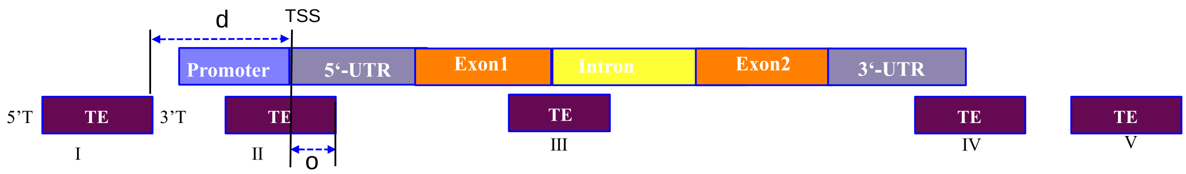

Categories of relationship between TE and flanking genes:

I. Upstream: 5'G > 3'T, d = 5'G – 3'T+1; if exist TSS: d = TSS-3'T, d <1000bp indicates promoter insertion

II. 5'-Overlap: 5'G < 3'T && 5'G > 5'T; o = 3'T – 5'G +1

III. Nested: 5'T > 5'G, 3'T < 3'G

IV. 3'-Overlap: 3'G > 5'T && 3'G < 3'T; o =3'G –5'T +1

V. Downstream: 3'G < 5'T; d = 5'T – 3'G + 1

For nested:
The two coordinates of un-dertermined TE elements were inserted into a sorted array, in which other coordinates of the features of targeted gene were stored in a hash table implemented in customer perl script and the spanned regions of the TE were determined by adjacent features. Exampled by a nested TE spanning the exon1 and adjacent intron:

(5'utr, 3'utr, 5'exon1, 5'T, 3'exon1, 5'intron, 3'T, 3'intron, 5'exon2, 3'exon2, 5'utr, 3'utr)

* the coordinates of Gene or TE represented by 5'G, 3'G, 5'T, 3'T, as other coordinates of gene features. TSS: transcripition start site

Figure S3. Schematic representation of the relationships between transposable elements and the nearest flanking host genes in the genome assembly of *Barthea barthei*. Five categories of relative relationships between the transposable elements and the nearest flanking genes were upstream, 5'-overlap, Nested, 3'-overlap, and downstream, respectively. The upstream and downstream of gene and transposable element marked as 5'G, 3'G, 5'T, 3T respectively.

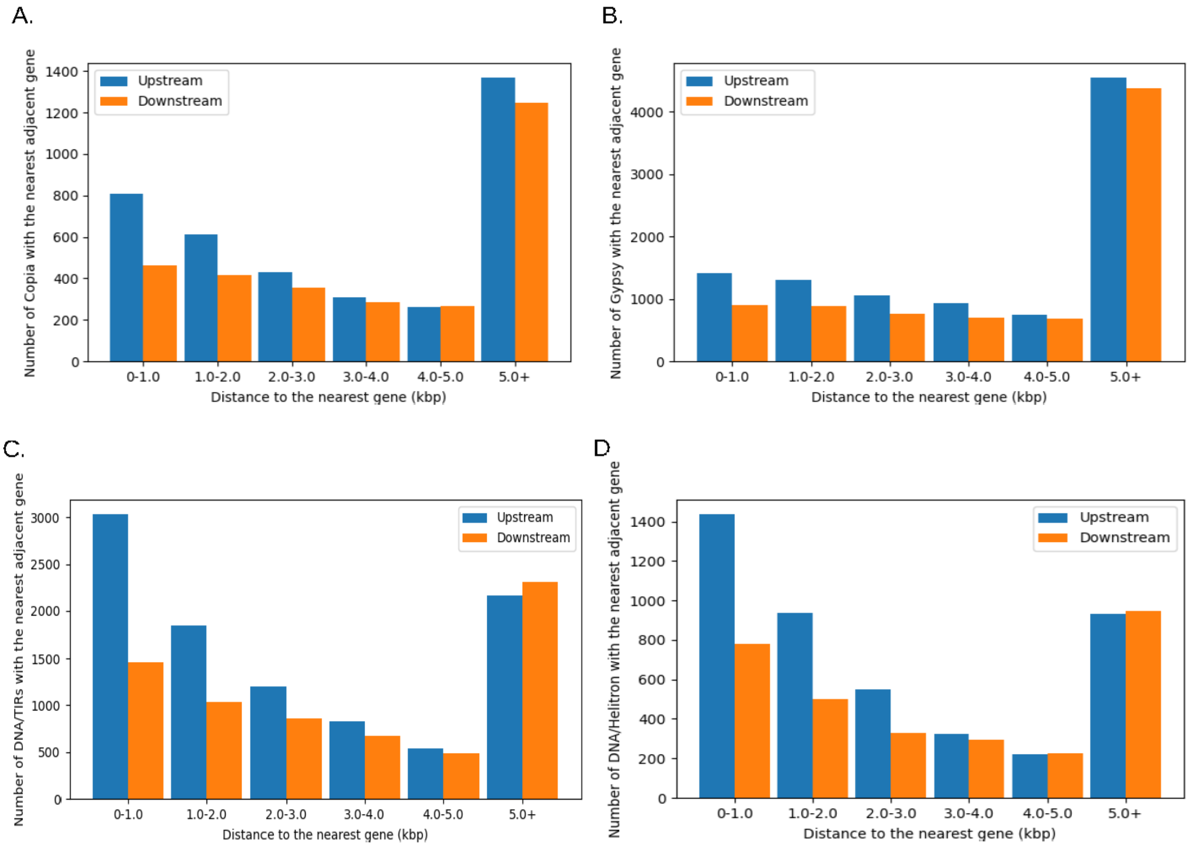

Figure S4. The distance distributions between the transposable elements and their nearest flanking host genes in the genome assembly of *Barthea barthei* for four categories of transposable elements, (A). LTR/Copia; (B). LTR/Gypsy; (C). DNA/TIRs; (D). DNA/Helitrons.

A.

B.

C.

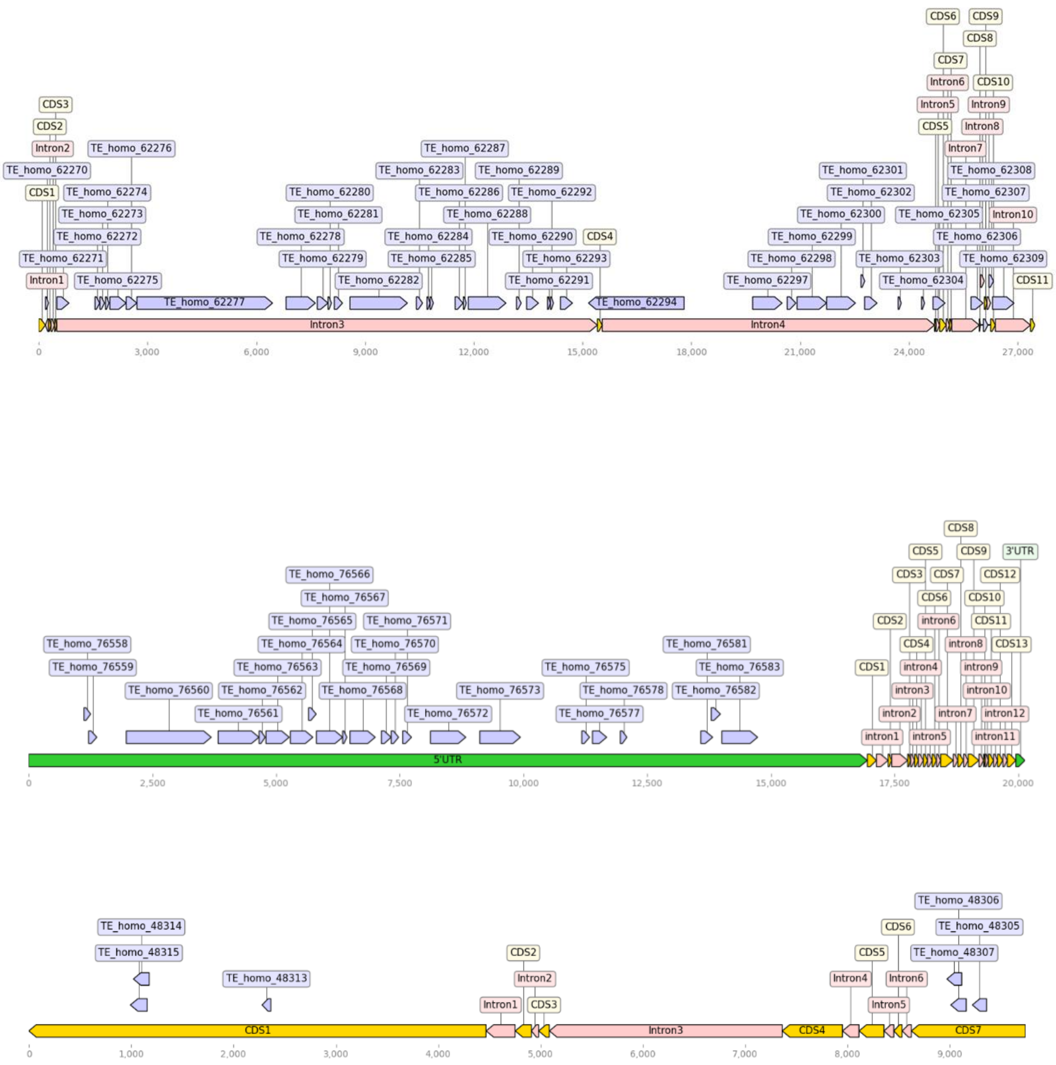

Figure S5. The Illustrations of different scenarios for host genes nested with distinct transposable elements in the genome assembly of *Barthea barthei*. (A). Gene *Barthea36295* nested with transposons in single intron3; (B). Gene *Barthea44534* nested with distinct transposons in the 5'UTR*;* (C). Gene *Barthea28554* nested with distinct transposons in the CDS region.

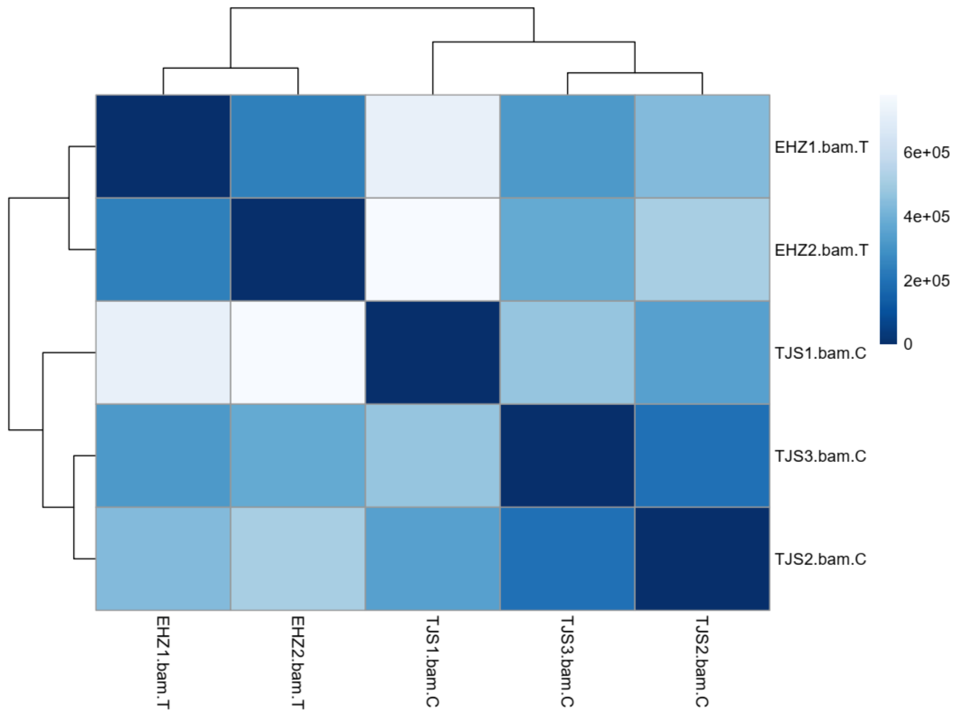

Figure S6. Heatmap of samples similarities based on Euclidean distances calculated using the normalized counts of gene/transposable element for the leaf transcriptomes of *Barthea barthei*.
